# Feline Leukemia Virus and Other Pathogens as Important Threats to the Survival of the Critically Endangered Iberian Lynx (*Lynx pardinus*)

**DOI:** 10.1371/journal.pone.0004744

**Published:** 2009-03-09

**Authors:** Marina L. Meli, Valentino Cattori, Fernando Martínez, Guillermo López, Astrid Vargas, Miguel A. Simón, Irene Zorrilla, Alvaro Muñoz, Francisco Palomares, Jose V. López-Bao, Josep Pastor, Ravi Tandon, Barbara Willi, Regina Hofmann-Lehmann, Hans Lutz

**Affiliations:** 1 Clinical Laboratory, Vetsuisse Faculty, University of Zurich, Zurich, Switzerland; 2 Programa de Conservación Ex Situ del Lince Ibérico, Espacio Natural de Doñana, Matalascañas, Spain; 3 Egmasa-Consejería de Medio Ambiente, Córdoba, Spain; 4 Centro de Análisis y Diagnóstico, Málaga, Spain; 5 Departamento de Biología de la Conservación, Estación Biológica de Doñana (CSIC), Sevilla, Spain; 6 Departament de Medicina i Cirurgia Animals, Facultat de Veterinària, Universitat Autònoma de Barcelona, Barcelona, Spain; Cambridge University, United Kingdom

## Abstract

**Background:**

The Iberian lynx (*Lynx pardinus*) is considered the most endangered felid species in the world. In order to save this species, the Spanish authorities implemented a captive breeding program recruiting lynxes from the wild. In this context, a retrospective survey on prevalence of selected feline pathogens in free-ranging lynxes was initiated.

**Methodology/ Principal Findings:**

We systematically analyzed the prevalence and importance of seven viral, one protozoan (*Cytauxzoon felis*), and several bacterial (e.g., hemotropic mycoplasma) infections in 77 of approximately 200 remaining free-ranging Iberian lynxes of the Doñana and Sierra Morena areas, in Southern Spain, between 2003 and 2007. With the exception of feline immunodeficiency virus (FIV), evidence of infection by all tested feline pathogens was found in Iberian lynxes. Fourteen lynxes were feline leukemia virus (FeLV) provirus-positive; eleven of these were antigenemic (FeLV p27 positive). All 14 animals tested negative for other viral infections. During a six-month period in 2007, six of the provirus-positive antigenemic lynxes died. Infection with FeLV but not with other infectious agents was associated with mortality (*p*<0.001). Sequencing of the FeLV surface glycoprotein gene revealed a common origin for ten of the eleven samples. The ten sequences were closely related to FeLV-A/61E, originally isolated from cats in the USA. Endogenous FeLV sequences were not detected.

**Conclusions/Significance:**

It was concluded that the FeLV infection most likely originated from domestic cats invading the lynx's habitats. Data available regarding the time frame, co-infections, and outcome of FeLV-infections suggest that, in contrast to the domestic cat, the FeLV strain affecting the lynxes in 2007 is highly virulent to this species. Our data argue strongly for vaccination of lynxes and domestic cats in and around lynx's habitats in order to prevent further spread of the virus as well as reduction the domestic cat population if the lynx population is to be maintained.

## Introduction

The Iberian lynx (*Lynx pardinus*) is native to the Iberian Peninsula and is considered the most endangered felid species in the world [1], [2], [3]. At present this species is especially threatened due to the decline of its basic prey (the wild European rabbit, *Oryctolagus cuniculus*), fragmentation and loss of its habitat, and non-natural mortality [4]. Iberian lynxes are confined to two isolated populations in southern Spain in the Doñana and Sierra Morena areas, and only 40–50 and 150–200, respectively, are estimated to remain [5], [6], [7]. To save this species from extinction, an EU LIFE Nature project is underway that includes habitat preservation, lynx population monitoring, and rabbit population management. In addition, cryopreservation of lynx genetic material and a captive *ex situ* breeding project were initiated to preserve the genetic diversity of the species and produce new specimens for future reintroduction. Despite extensive information concerning the field ecology of this species [8], [9], [10], [11], [12], [13], only limited information is available regarding the diseases that affect the Iberian lynx in the wild or in captivity. Infections due to *Cytauxzoon felis* (a Theileria-like agent), *Toxoplasma gondii*, hemotropic mycoplasmas, *Mycobacterium bovis*, and several parasitic diseases have been described in some individuals [14], [15], [16], [17], [18], [19], [20], [21]. Bovine tuberculosis was reported as the cause of death for five Iberian lynxes between 1998 and 2007 [22], [23], [24], [25], [26], [27]. Histopathological studies revealed generalized immune depletion in these animals, apparently unrelated to infectious agents or malnutrition [24], and glomerulonephritis [28]. Furthermore, the presence of feline leukemia virus (FeLV) provirus was recently reported in six samples originating from both the Doñana and Sierra Morena areas in southern Spain between 1994 and 2003 [29].

A recent study documented the limited genetic diversity of the Iberian lynx population [30]. Similarly to the cheetahs, where limited genetic diversity had been shown to be associated with disease by an otherwise harmless coronavirus [31], [32], the restricted genetic basis of Iberian lynxes may contribute to render this endangered species particularly susceptible to pathogens and possibly even to opportunistic infectious agents. Therefore, a retrospective study, aimed to systematically analyze the prevalence and importance of known feline pathogens in free-ranging Iberian lynxes, was initiated. Animals were screened for the following viral agents: feline herpesvirus (FVH), feline calicivirus (FCV), feline parvovirus (FPV), feline coronavirus (FCoV), feline leukemia virus (FeLV), feline immunodeficiency virus (FIV), and canine distemper virus (CDV). These viral infections are known to affect also wild felids [33], [34], [35]. In addition, animals were tested for *C. felis*, *Anaplasma phagocytophilum*, hemotropic mycoplasmas, *Bartonella henselae*, and *Chlampydophila felis*. Thus, in the present study, we report on the prevalence of the aforementioned pathogens and we describe a dramatic FeLV epidemic, which most likely led to the death of 6 Iberian lynxes within a 6-months period in 2007, its possible origin, and its relationship to other infectious agents. As a consequence of this study, we hope to provide insight on how to reduce danger originating by these infections and to raise public awareness about the critical situation of the Iberian lynx.

## Results

### Case history and clinical manifestations

**Figure 1:**
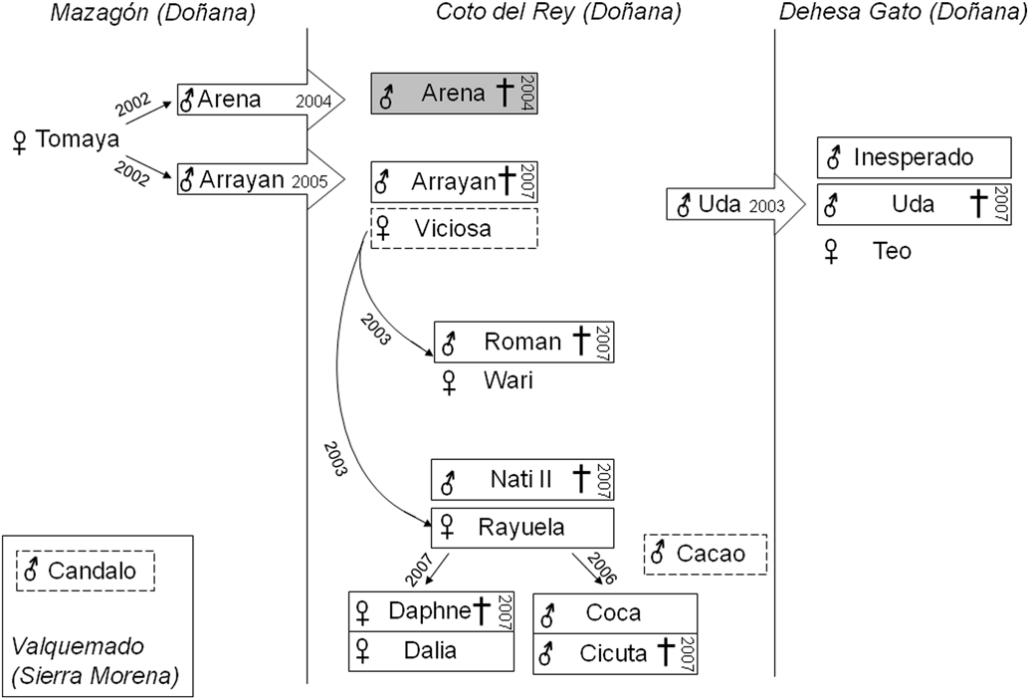
Overview of the geographical distribution and relationships between FeLV-infected lynxes. The regions of Mazagón, Coto del Rey and Dehesa Gato (Doñana National Park), and of Valquemado (Sierra Morena) are depicted schematically. All animals are identified by names. Large open arrows indicate migration after birth, accompanied by the year of migration. Symbols indicate females and males. Dead animals are indicated by a cross (†) accompanied by the year of death. Small arrows indicate descendants of the females, and the birth year is also indicated. Frames with dashed lines indicate FeLV-infected animals; frames with continuous lines indicate FeLV-infected animals from which the FeLV *env* genes were sequenced. Gray shading (animal “Arena”) indicates the FeLV variant most distantly related to the other FeLV found in these lynxes. The names of lynxes having close contact are positioned in close vicinity.

Sixteen of the 77 Iberian lynxes studied died. Seven lynxes were found dead after being stuck by vehicles, one due to illegal hunting, and eight died for reasons presumed to be related to infectious diseases. One of the latter eight lynxes exhibited high viral loads of CDV, suggesting that CDV infection was the cause of death (data not shown). In another, young animal, *M. bovis* was found in the tracheal wash. One of the animals struck by a vehicle had been found to be FeLV provirus-positive in 2004. The remaining six dead lynxes were all positive for FeLV provirus and FeLV p27 antigen, and all died within six months during the spring-summer of 2007 in the northern part of the Doñana area (Fig. 1).

In a routine check of animals (n = 16) in this area in early December 2006, an adult free-ranging male was found to be FeLV provirus- and p27-positive. The same animal was negative for FeLV when sampled one year before. This animal demonstrated no clinical signs and was released. In May 2007, the same lynx was found dead. In December 2006, the other 15 lynxes from the northern part of the Doñana area were FeLV-negative. Beginning in March 2007, five of these 15 lynxes died: all five were positive for FeLV provirus and FeLV p27. In the same period additional seven lynxes were found to be FeLV-positive and survived. FeLV p27-positive animals were captured and transported to the Los Villares rescue center in order to reduce infectious pressure in the field [36]. Relationships and geographical distribution of the infected lynxes are depicted in Figure 1. The majority of the remaining lynxes (n = 61) were clinically healthy.

### FeLV infection is associated with increased mortality of Iberian lynxes

To determine the association of FeLV infection with mortality, the proportions of FeLV infection in dead and surviving lynxes were analyzed for significant differences by Fisher's exact test. Both, provirus positivity and antigenemia, were significantly correlated with mortality (*p*_Fisher_ = 0.0138 for provirus; *p*_Fisher_ = 0.0187 for antigenemia). The significance level even increased when the traumatic deaths (n = 8) were omitted from the comparison (*p*_Fisher_ = 0.0007 for provirus; *p*_Fisher_ = 0.0002 for antigenemia). Provirus positivity was less frequent in lynxes that died from traumatic reasons than in the total population of the Doñana area (*p*_Fisher_ = 0.041).

### Endogenous FeLV sequences

The quantitative real-time PCRs specific for endogenous FeLV sequences were weakly positive in samples obtained from 5 of 77 lynxes. Four animals tested positive in only one of the three assays used, one was positive for the enU3-1, enU3-2 and env assays. However, the enFeLV copy numbers were extremely low, with less than 1 copy per 10'000 cells, suggesting that the weak positive results may be the consequence of sequences cross reacting with the assay.

### FeLV and its possible origin in lynxes

**Figure 2:**
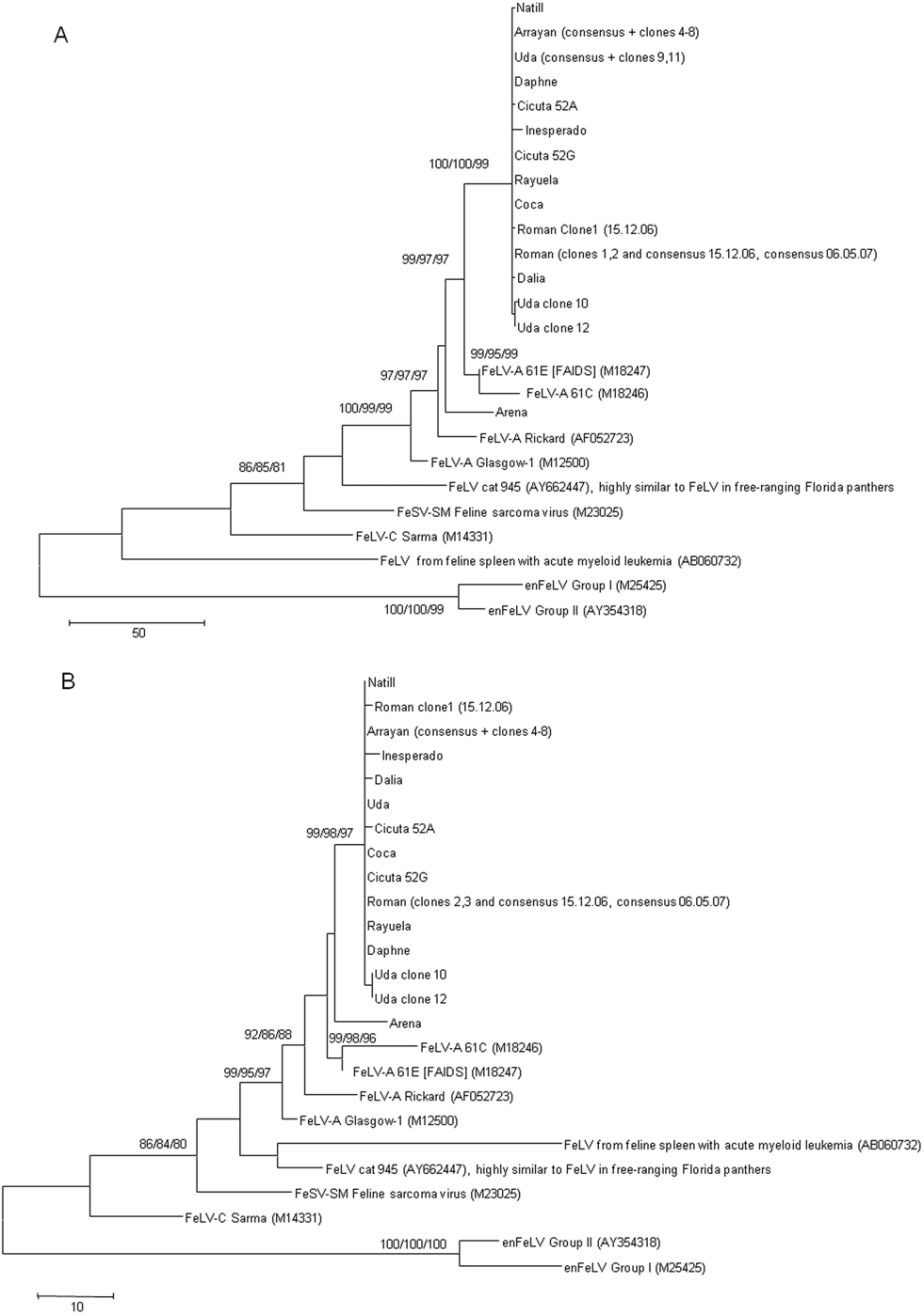
Evolutionary relationships of Iberian lynx FeLV SU. The Maximum Parsimony (MP) tree is shown. Trees are drawn to scale; length is in terms of the number of changes over the entire sequence. (A) Relationships at the DNA level. MP tree length = 643, consistency index = (0.744726), retention index = (0.827143), composite index = 0.671491 (0.615995) for all sites and parsimony-informative sites (in parentheses). The codon positions included were 1st+2nd+3rd+Noncoding. There were a total of 1424 base positions in the final dataset, of which 311 were parsimony-informative. (B) Relationships at the protein level. MP tree length = 253, consistency index = (0.861538), retention index = (0.876712), composite index = 0.783150 (0.755321) for all sites and parsimony-informative sites (in parentheses). There were a total of 473 amino acid positions in the final dataset, of which 108 were parsimony informative.

Fourteen lynxes (13 from the Doñana area and one from Sierra Morena) tested positive for FeLV-A provirus (Table 1), but negative for FeLV-B and FeLV-C (data not shown). FeLV provirus was amplified and sequenced from eleven positive lynxes from the Doñana area. Nucleotide sequence analysis of the surface unit (SU) of the *env* gene revealed a common origin for the provirus found in Doñana lynxes in 2007 (99.5–100% identity). The sequences clustered with and were 97.9–98.2% identical to the FeLV-A/61E strain (Fig. 2A) [37], [38], while the sequence obtained from the lynx found to be provirus-positive in 2004 (Arena) was only 94.8–95% identical to the other sequences and was more related to FeLV-A/Rickard (97.4% identity, Fig. 2A). The predicted amino acid sequences of the SU glycoprotein from animals infected in 2007 presented an identity/similarity of 97.3–97.6%/98.6–98.9% to FeLV-A/61E and 95.9–96.2%/97.6–97.8% to FeLV-A/Rickard, while the identity/similarity of the sequence from the animal infected in 2004 was 97.3%/98.6% to FeLV-A/61E, 96.2%/97.8% to FeLV-A/Rickard, and 96.7–97.0%/98.9–99.2% to the 2007 sequences. At the protein level, all sequences from the Iberian lynxes clustered together with FeLV-A/61E and FeLV-A/Rickard (Fig. 2B). No sequences showing mutations that could suggest a close relationship to FeLV-A/61C were found.

**Table 1:** Overview of the prevalence of infection in Iberian lynxes.

	Doñana National Park				Sierra Morena			
	Serology		PCR		Serology		PCR	
	Pos/Total	%	Pos/Total	%	Pos/Total	%	Pos/Total	%
**Viral infections**								
FeLV	11/45*	24.4*	13/45	28.9	0/30*	0*	1/32	3.1
FIV	0/44	0	0/45	0	0/30	0	0/32	0
FCoV	7/44	15.9	0/45	0	12/30	40	0/32	0
FHV	7/44	15.9	0/45	0	2/30	6.7	0/30	0
FPV	13/44	29.5	2/45	4.4	9/30	30	0/30	0
FCV	15/44	34.1	0/45	0	14/30	46.7	0/30	0
CDV	11/44	25	1/45	2.2	1/30	3.3	0/30	0
**Protozoan infections**								
*Cytauxzoon felis*	n.a.	n.a.	0/45	0	n.a.	n.a.	24/32	75
**Bacterial infections**								
*M. haemofelis*	n.a.	n.a.	16/45	35.6	n.a.	n.a.	9/32	28.1
*C.*M. haemominutum	n.a.	n.a.	13/45	28.9	n.a.	n.a.	14/32	43.8
*C.*M. turicensis	n.a.	n.a.	6/45	13.3	n.a.	n.a.	4/32	12.5
*A. phagocytophilum*	1/44	2.3	0/45	0	3/30	10	0/30	0
*B. henselae*	n.a.	n.a.	6/45	13.3	n.a.	n.a.	10/30	33.3
*Chlamydopila felis*	n.a.	n.a.	0/45	0	n.a.	n.a.	1/30	3.3

FeLV: feline leukemia virus; FIV: feline immunodeficiency virus; FCoV: feline coronavirus; FHV: feline herpesvirus; FPV: feline parvovirus; FCV: feline calicivirus; CDV: canine distemper virus; M. = Mycoplasma; C.M. = Candidatus Mycoplasma; A. = Anaplasma; B. = Bartonella; * = p27 antigen; n.a. = not applicable.

### Prevalence of other infections

Antibodies to FCV were detected in 29/74 (39.2%), to FPV in 22/74 (29.7%), to FCoV in 19/74 (25.7%), to CDV in 12/74 (16.2%), to FHV in 9/74 (12.2%), and to *A. phagocytophilum* in 4/74 (5.4%) animals (Table 1). Antibodies to FIV were not detected in any of the 74 samples tested. Eleven out of 75 lynxes tested positive for FeLV p27 antigen. All FeLV antigenemic lynxes originated from the Doñana area. PCR of blood samples detected FeLV in 14/77 (18.2%), FPV in 2/75 (2.7%), CDV in 1/75 (1.3%), *C. felis* in 24/77 (31.2%), *M. haemofelis* in 25/77 (32.5%), ‘*Candidatus* M. haemominutum’ in 27/77 (35.1%), ‘*Candidatus* M. turicensis’ in 10/77 (13%), *Ch. felis* in 1/75 (1.3%), and *B. henselae* in 16/75 (21.3%) samples. CDV- and FPV-positive animals were found only in the Doñana area, while all *C. felis*-positive lynxes originated from Sierra Morena. PCR of all blood samples did not detect FIV, FCoV, FHV, FCV, or *A. phagocytophilum*. The detailed results are compiled in Table 1. Fecal samples tested negative for FCoV (0/68) and positive for FPV in 1/62 (1.6%) and CDV in 1/45 (2.2%) samples; the latter was obtained from the only animal in which CDV was found in the blood. Only six fecal samples from the 14 FeLV provirus-positive animals were available. Five fecal samples originating from antigenemic animals tested positive for FeLV DNA, whereas one sample from a p27-negative animal tested negative by PCR (data not shown).

### Association between FeLV and other infectious agents

Statistically significant associations (based on PCR results) were found between FeLV infection and infections with *M. haemofelis* (*p*_Fisher_ = 0.0004) and ‘*Candidatus* M. turicensis’ (*p*_Fisher_ = 0.0205). The other pathogens did not show any significant association with FeLV infection.

## Discussion

In view of the endangered situation of the Iberian lynx, the central government of Spain in conjunction with the autonomous government of Andalusia has initiated an *ex situ* conservation program comprising captive breeding centers. Animals that were selected as founders for the captive breeding project had been caught in the wild and tested for the absence of pathogens prior to introduction into the captive breeding program. Of note, some of the founder lynxes were found to be infected with *C. felis*, hemotropic mycoplasmas and *B. henselae*, and also to exhibit antibodies to other infectious agents. However, these animals were clinically healthy. Between late 2003 and 2007, substantial information was obtained regarding the prevalence and importance of infectious agents in Iberian lynx populations of the Doñana and Sierra Morena habitats. With the exception of FIV, evidence for all tested feline pathogens was found. Interestingly, the seroprevalence of FHV, FCV, FPV, and FCoV was substantially higher than that observed in free-ranging Eurasian lynxes (*Lynx lynx*) from Sweden [39]. The increased seroprevalence of these feline pathogens in Iberian lynxes probably reflects higher population density and closer contact with domestic cats as compared to lynxes in the Swedish population. It is clearly desirable that the infectious pressure by these feline pathogens arising from domestic cats should be kept as low as possible.

Before the fall of 2006, FeLV infection (provirus) had been detected in one animal (“Arena”) in 2004. In a recently published retrospective study [29], six of 21 lynxes sampled between 1993 and 2003 were found to be FeLV provirus-positive, but not antigenemic. Although no clinical data were reported in that study, there was no indication that FeLV infection would represent a major risk for the lynx population. This situation changed acutely when, in 2007, several animals with severe clinical signs were found to be infected with FeLV and eventually died, the statistical association between FeLV infection and death being highly significant. Interestingly, presence of FeLV-provirus in lynxes that died from traumatic incidents (1 shot, 7 hit by cars) was significantly lower than in the lynxes with FeLV infection found dead. From this it was concluded that FeLV provirus positivity was not a predisposing factor for death by accidents. The pathogenicity of the FeLV strain involved in the outbreak, with six of 13 infected animals succumbing within six months, was rather unexpected. Five of the FeLV-infected lynxes that had died were living in the Coto del Rey nucleus, where three adult pairs of lynxes and their offspring normally only use 700–1000 ha [13]. Consequently, contact between individuals may be frequent in this area [40]. The other FeLV-positive dead lynx was living in the Gato nucleus, where animals have relatively high rates of contact with those living in the Coto del Rey nucleus via dispersal [9]. Several of the FeLV-infected lynxes showed clinical signs and/or hematologic abnormalities, such as anemia, lymphopenia or neutropenia, compatible with FeLV infection also observed in domestic cats. In contrast, the rapid onset of the clinical signs leading to death are not a typical feature of FeLV-A infection in cats, where some FeLV infected animals can survive more than 10 years [41], [42]. The presence of co-infections could also have contributed to the high pathogenicity of FeLV in the lynxes. From the observation that a significant association exists between FeLV and hemotropic mycoplasma infections, one is tempted to argue that these co-infections may lead to increased pathogenicity. However, although the association is statistically significant, co-infection by FeLV and hemotropic mycoplasms cannot be the cause of death in all cases, as all lynxes that survived FeLV infection were also positive for hemotropic mycoplasms. It is possible that pre-existing infection by these agents may favor FeLV infection. The increased frequency of FeLV infection in lynxes positive for hemotropic mycoplasms could also reflect simultaneous transmission of these agents with FeLV during cat-to-lynx contact.

Sequencing of the complete FeLV *env* gene revealed that the virus present in lynxes affected in 2007 was clearly distinct from the virus detected in the FeLV-positive lynx found dead in 2004. This suggests that FeLV was introduced into the Iberian lynx population on at least two occasions. Nonetheless, all analyzed FeLV sequences were closely related to FeLV-A and, in particular, to FeLV-A/61E, a member of a highly conserved family representing prototype viruses of the horizontally transmitted, minimally pathogenic FeLV-A. These FeLV-A prototype viruses are assumed to be present in all naturally occurring FeLV infections in domestic cats. FeLV-A/61E in conjunction with FeLV-A/61C was found to cause severe immunodeficiency in domestic cats (FeLV-FAIDS) and was originally isolated from a cat in Colorado [43]. Although we were not able to detect envelope variants similar to FeLV-A/61C in the blood, the presence of additional FeLV variants in different organs cannot be excluded. Thus, important minority genomes harboring changes in the *env* gene sequence may have been present but were not detected. To exclude this possibility, we not only sequenced the majority of FeLV variants present in the peripheral blood, but also cloned FeLV from four lynxes and analyzed several clones from each animal, without finding additional sequences. In domestic cats with FeLV-FAIDS, the second virus variant (FeLV-A/61C) was mainly found in the intestines and was not present in the blood [37]. Thus, further investigations should include such tissue samples from infected lynxes. Alternatively, mutations, deletions, or repeats in other parts of the FeLV genome that were not characterized in the present study (e.g., in the LTR) could have contributed to the pathogenicity of the virus. FeLV variants with 21-bp tandem repeats in the LTR have been isolated from domestic cats with naturally occurring lymphomas and, more recently, from Florida Panthers [44], [45], [46]; the LTR transcriptional enhancer repeats were described in T-cell tumors [47]. These 21-bp repeats confer a replicative advantage to the virus rather than modify the disease spectrum [44], [47]; they were not investigated in the present study.

Furthermore, the high pathogenicity observed in FeLV-infected Iberian lynxes might be due to the host, rather than solely to viral factors. The genetic diversity of the Doñana lynx population is lower than that of the Sierra Morena population [30], and therefore the pathogenic potential of FeLV could have been enhanced by inbreeding. In addition, an immune-mediated systemic disease of unknown origin has been recently postulated [28]. Cheetahs, another wild felid species with reduced genetic diversity, also demonstrate increased susceptibility to some infectious disease agents [48]. Endogenous FeLV-related sequences (enFeLV) could be another host factor that might contribute to the high pathogenicity of FeLV in lynxes [49], [50]. These enFeLV exist as multiple, nearly full-length proviral sequences (1 to 100 copies/cell) in domestic cats, as well as in closely related wild cats of the genus *Felis*, e.g., European wildcats (*Felis silvestris silvestris*) [51]. However, endogenous FeLV sequences related to those of domestic cats are apparently not present in Iberian lynxes: only 5 of the 77 lynxes tested displayed weak signals by quantitative real-time PCR, which is not compatible with presence of enFeLV sequences. As endogenous retroviruses are integrated in the germ line and by consequence should be present in at least one copy per cell, the results obtained can only be explained by the presence of either endogenous sequences distantly related to the enFeLV of domestic cats or of a co-infection with another gammaretrovirus, both showing a weak cross-reactivity to the real-time PCR systems used. The absence of FeLV-B, which is the result of a recombination between exogenous and endogenous FeLV, is also in agreement with the absence of enFeLV in the Iberian lynxes, since the incidence of FeLV-B in the domestic cat is usually higher than 50% in diseased cats [52]. Absence of enFeLV sequences suggests that the mechanisms inducing disease in lynxes must be distinct from those in FeLV-B-infected cats.

As only a few cases of FeLV infection were observed prior 2007 [29], it is likely that FeLV infection in lynxes is rare, is not readily carried within the lynx population, and most likely originates from domestic cats, which are increasingly frequent in Doñana. A FeLV prevalence of 15.6% was reported in Spanish cats [53]; this might be even higher around Doñana (F. Martinez, personal communication). Therefore, a higher risk of transmission from domestic cats to lynxes is likely. Since some FeLV variants seem to be highly pathogenic in the Iberian lynx, it would seem important for the survival of lynxes to protect them against further FeLV transmission from domestic cats. Thus, it is important that FeLV infection in free-ranging lynxes be controlled as quickly as possible. To this end, conservation authorities have started to vaccinate lynxes against FeLV infection using a Canarypox-based FeLV vaccine [54]. From what is known about the control of FeLV infection in domestic cats, vaccination is highly effective. Although vaccination did not induce sterilizing immunity in domestic cats, it was nonetheless able to stimulate the immune system to a degree that allowed the cats to overcome the infection rapidly and to clear most of the viral RNA from the blood [55]. Even if immunization does not completely protect lynxes against transient infection, there is justified hope that the outcome of FeLV infection will be less severe. In addition to vaccination, it is probably also important to decrease the infectious pressure on lynxes by preventing domestic cats from entering the lynx habitats and by vaccinating them against FeLV infection to reduce the risk of FeLV transmission to lynxes.

## Materials and Methods

### Animals and samples

From late 2003 until September 2007, EDTA-anticoagulated blood, serum, and fecal samples from 77 free-ranging Iberian lynxes were collected in both the Doñana (n = 45) and Sierra Morena (n = 32) areas in the South of Spain. Some of these animals were later included in the *ex situ* breeding program. Only samples collected at the time of capture (free-ranging status) were included in this study. Blood samples were collected from the *Vena cephalica* of animals that had been caught and immobilized with a mixture of ketamine-medetomidine for radiocollaring, sanitary checking, allocation to the captive breeding, or other management programs. Blood or serosanguineous samples were also collected from the thoracic cavity or the heart of animals found dead.

### Nucleic acid extraction from blood, fecal, and tissue samples

Total nucleic acids (TNA) were extracted from 200 µl of EDTA-blood, serosanguineous fluid, or fecal samples using the MagNA Pure LC Total Nucleic Acid Isolation Kit (Roche Diagnostics, Rotkreuz, Switzerland), or from tissues collected upon necropsy using DNeasy and/or RNeasy Tissue kits (Qiagen, Hombrechtikon, Switzerland) according to the manufacturer's instructions.

### Detection of specific infections

#### Serological methods

Serum samples from 75 animals were available for the serological assays. The presence of antibodies against FHV, FCV, FPV, FCoV, and CDV was determined by an immunofluorescence assay (IFA), and antibodies to FIV were detected by ELISA and western blot, as previously described [33], [56]. FeLV p27 antigen was detected and quantified by ELISA as previously described [57]. Antibodies against *A. phagocytophilum* were determined by an immunofluorescence assay using commercially available IFA slides (VMRD, Inc. Pullman, WA, USA).

#### Detection and quantification of DNA and RNA

DNA specific for FHV, FPV, FIV, and FeLV provirus, *B. henselae*, *Ch. felis*, *A. phagocytophilum*, and hemotropic mycoplasmas was detected and quantified by real-time TaqMan PCR, as previously described [58], [59], [60], [61], [62], [63], [64], [65], [66]. The presence of *C. felis* was detected by conventional PCR using the forward primer Cytfelis.203f (5′-AGA CCY YAA ACC ATC CCG CT-3′) and reverse primer Cytfelis.423r (5′-CCT GCT GCC TTC CTT AGA TG-3′) (Microsynth, Balgach, Switzerland). The PCR reactions contained 2.5 units of Taq DNA Polymerase (Sigma, Buchs, Switzerland), a final concentration of 500 nM of each primer, 200 µM dNTPs (Sigma), 2.5 µl of 10× PCR Buffer (Sigma), and 2.5 µl of template in a final volume of 25 µl. An initial denaturation of 5 min at 95°C was followed by 35 cycles of 95°C for 30 s, 70°C for 45 s, and 72°C for 1 min, with a final extension at 72°C for 10 min on a Biometra TPersonal thermal cycler (Biolabo, Chatel-St-Denis, Switzerland).

RNA specific for FCV and FCoV was detected and quantified by real-time TaqMan RT-PCR, as previously described [59], [67], [68]; for CDV the RT-PCR reaction was set up with 12.5 µl of One-step RT qPCR Mastermix Plus (Eurogentec, Seraing, Belgium), as well as a final concentration of 600 nM each for the forward primer CDV.78f (5′-GGA AGC CTT GAT GAT AGC ACT GA-3′) and reverse primer CDV.161r (5′-GCC GAA AGA ATA TCC CCA GTT-3′, Microsynth) and 200 nM for the fluorogenic probe CDVp (5′-6FAM- TCT GGC GAA GAT TAT TCC GAA GGA AAT GCT-TAMRA-3′, Eurogentec). Finally, 5 µl of RNA was used in a 25 µl total reaction volume. Reverse transcription for 30 min at 48°C was followed by a denaturation step at 95°C for 10 min and 45 cycles of 95°C for 15 s and 60°C for 30 s. Real-time PCR and RT-PCR were performed using an ABI Prism 7700 sequence detection system (Applied Biosystems, Rotkreuz, Switzerland).

### PCR assays used to characterize FeLV infection

#### Specific FeLV-B and –C subtype detection

FeLV-B was detected as previously described [69]. For FeLV-C, PCR was designed to amplify a 2 kb region encoding the envelope and the 3′LTR of FeLV-C. The specificity of the system was validated as described for FeLV-B [69]. The PCR reactions contained 0.4 units of Phusion™ High Fidelity DNA Polymerase (Finnzymes, Keilaranta, Finland), final concentrations of 500 nM each for the forward primer 5944F and reverse primer 8041R (Table 2), 200 nM dNTPs (Sigma), 4 µl 5× PCR HF Buffer (Finnzymes), and 5 µl of template in a final volume of 20 µl. An initial denaturation of 30 s at 98°C was followed by 35 cycles of 98°C for 20 s, 64°C for 30 s, 72°C for 2 min, and a final extension at 72°C for 5 min on a Biometra TPersonal thermal cycler (Biolabo).

**Table 2:** PCR primers used for amplification and sequencing of FeLV *env* and for detection of FeLV-B and -C subtypes.

Name	Sequence 5′-3′	Length	Ref.
5617F	CCTATGGCTCACTTCTTTGATACTGATATCTCTA	34	This paper
5847F	ACATATCGTCCTCCTGACCAC	21	[79]
5944F	GATCAGGACCTCCCGAACGACCCT	24	[79]
6124F	GTAATAACCAATATGCAAACTAACACCC	28	This paper
6124eF	ACAATAACCAACCTTGTAACTGGAACAA	28	This paper
6124R	GGGTGTTAGTTTGCATATTGGTTATTAC	28	This paper
6382F	TGGGGCCAAAGGGAACACAT	20	This paper
6382R	ATGTGTTCCCTTTGGCCCCA	20	This paper
6956F	CGAAGGGATTGCAATCTTAGGTAAC	25	This paper
6956R	GTTACCTAAGATTGCAATCCCTTCG	25	This paper
7266R	TGTGTACACATATTCGGGTTGATGGTA	27	This paper
8041R	CCTAACTTCCTTATATCTCATGGGAACATGT	31	This paper
8197R	GAAGGTCGAACCCTGGTCAACT	22	[79]

All primers were synthesized by Microsynth, Balgach, Switzerland.

#### Full-length FeLV-A *env* amplification, subcloning, and sequencing

TNA from blood or DNA from tissue samples was used for amplification of the complete FeLV provirus *env* gene. To discover specific primers, a multiple sequence alignment (MSA) of all GenBank FeLV *env* sequences (available as of June 2006) was compared with the MSA of endogenous FeLV (enFeLV) and FeLV-C *env* entries. Primers that anneal to FeLV-A, but not enFeLV or FeLV-C, were used (Table 2). MSAs were constructed using the multiple alignment editor Jalview [70]. The primer melting temperature and degree of self-annealing were determined using the GCG software package (Accelrys, San Diego, CA, USA). For amplification of full *env*, PCR reactions comprised 0.4 units of Phusion™ High-Fidelity DNA Polymerase (Finnzymes), final concentrations of 500 nM each for primers 5617F and 8197R, 200 µM dNTPs, 4 µl of 5× Phusion™ HF Buffer, and 2 µl of template in a final volume of 20 µl. PCR was performed using a Biometra Tpersonal PCR thermal cycler (Biolabo). After an initial denaturation at 98°C for 30 s, amplification was performed using 35 cycles of 98°C for 20 s, 64°C for 30 s, and 72°C for 2 min 30 s, and a final extension of 72°C for 8 min. PCR products were either sequenced directly after purification using the GenElute PCR Clean-Up Kit (Sigma) or separated by agarose gel electrophoresis, excised from the gel, purified using the GenElute Gel Extraction Kit (Sigma), and cloned into the pCRII-TOPO vector (Invitrogen, Basel, Switzerland) according to the manufacturer's instructions; the obtained clones were then sequenced. Sequencing was carried out by Synergene Biotech, Zurich, Switzerland using primers 5617F, 5847F, 6123F, 6123R, 6382F, 6382R, 6956F, 6956R, 7266R, and 8197R (Table 2). The resulting Iberian lynx FeLV-A *env* surface glycoprotein (SU) sequences were deposited in the GenBank database under accession numbers EU293175 to EU293194. MSAs of Iberian lynx enFeLV and FeLV subtypes *env* SU (GenBank Accession Numbers: M18247, M18246, M12500, AF052723, M14331, AY662447, AB060732, M23025, M25425, AY354318) were constructed using ClustalW [71] and refined using T-Coffee [72]. Phylogenetic trees were constructed using MEGA3 Software [73]. Boostrap support (% of 1,000 bootstrap replicates) was calculated using the neighbor-joining, minimum evolution, and maximum parsimony methods.

#### Endogenous FeLV (enFeLV) detection and quantification

The enFeLV DNA loads were determined as described using three different systems (enU3-1, enU3-2, and enEnv) [51] that detect enFeLV sequences present in the recently annotated cat genome [74]. A feline glyceraldehyde-3-phosphate dehydrogenase (fGAPDH) pseudogene, of which one copy is present in the genomic DNA of feline cells [64], was quantified by real-time TaqMan PCR assay [75]. Copy numbers of enFeLV were divided by fGAPDH copy numbers to calculate the number of copies per cell.

### Phylogenetic analyses

Phylogenetic trees were constructed using MEGA3 [73]. Bootstrap support (1000 replicates) was calculated by the neighbor-joining/minimum evolution/MP methods and considered significant when >70% [76]. MP trees were obtained using the Close-Neighbour-Interchange algorithm [77] with search level 3 [76], in which initial trees were obtained by random addition of sequences (10 replicates). All alignment gaps were treated as missing data. Branch lengths were calculated using the average pathway method [77].

### PID (percent identity/similarity)

PID values for DNA and predicted protein sequences were calculated using the software package MatGAT (Matrix Global Alignment Tool) version 2.02 [78].

### Statistical analyses

To assess associations between FeLV and other infectious agents, infection frequencies were compared using the Fisher's exact test (GraphPad Prism version 3.00 for Windows, GraphPad Software, San Diego, USA). P-values<0.05 were considered as significant. Group comparison was performed using Fisher's exact test (Analyse-it for Microsoft excel version 2.03, Analyse-it Software, Ltd., Leeds, UK).
